# 

**Published:** 1876-04

**Authors:** 


### TABLE OF CONTENTS.

ORIGINAL COMMUNICATIONS.

IS ELECTRICITY AS A THERAPEUTIC A HUMBUG? *By James B. Baird, M.D., Atlanta, Ga.* .......... 1
TWO CASES OF BI-LATERAL LITHOTOMY. *By John S. Coleman, M.D., Augusta, Ga.* .......... 10

ATLANTA ACADEMY OF MEDICINE.

Contraction of Muscles of Neck, 12; Decoction of Chestnut Leaves in Whooping Cough, 14; Knock-Knee, with Remarks, 15; Maggots in Eye, 17; Hemorrhoids, 17; Articular Rheumatism, 17; Epilepsy, 18; Rupture of Membranes .......... 19

CLINIC ATLANTA MEDICAL COLLEGE.

Services of A. W. Calhoun, M.D., 20; Ciliary Neuralgia, Cure by Quinine, 20; Sympathetic Inflammation of Eye, 21; Suppurative Inflammation of Middle Ear, with Necrosis of Mastoid Process, 22; Traumatic Cataract, with Iritis and Secondary Cataract.... 24
Notes, by W. S. Kendrick, M.D., for Examination of Class of Atlanta Medical College.......... 26

OUR EXCHANGES.

Potts' Disease of the Spine, 31; Lime Water in Infantile Eczema, 32; Deaths from Nervous Diseases, 33; Cough, 34; Diphtheria, 35; Common Membranous Sore Throat, 36; New Method of Preventing Secretion of Milk in Female Breast, 37; Bacteria Found in Perspiration of Man, 38; Treatment of Constipation and Diarrhœa, 39; Trenton Medical Association, 39; Russian Cure for Drunkenness, 41; Abscess of Anus in Typhoid Fever, 41; Petroleum in Skin Diseases, 42; Sulphite of Soda in Acute Dysentery, 42; Cure of Ovarion Tumors by Electrolysis, 43; Prevention of Pain after actual Cautery, 43; Extirpation of Larynx and Part of Tongue, 44; Salicylic Acid in Foul Breath, 45; Agreeable mode of taking Castor Oil, 46; Poisoning by Chloral – Antidote, 46; Goitre in Liberia, 47; Results of Operations on Scrofulous Persons, 47; Ergot—Its Active Principles, 48; Pathology of Hydrophobia, 49; Uterine Hemorrhage, 50; Digitalis in Bronchitis, 51; Treatment of Chronic Dysentery, 52; Specula for Examing Female Bladder, 53; Treatment of Ozœna, 54; Calculi in Prepuce of Child, 54; Lunacy in Town and Country, 55; Case of Suppurative Pericarditis, 55; Compression in Acute Rheumatism, 55; Radical Cure for Piles.......... 56

BIBLIOGRAPHICAL.

Treatise on Surgery.......... 56
System of Midwifery .......... 57
Disease of Infancy and Childhood .......... 57
Phthisis .......... 58
Extra-Uterine Pregnancy.......... 58

Gleanings .......... 58

EDITORIAL.

International Medical Congress.......... 62
Death of Dr. Nottingham.......... 63
Medical Association of Georgia.......... 64

